# Colorectal Cancer Risk Loci: Prognostic Factors for Clinical Outcomes? A Systematic Review and Meta‐Analysis

**DOI:** 10.1002/cnr2.70230

**Published:** 2025-05-19

**Authors:** Chengmi Wu, Jingyi Zhou, Qian Wu, Shu Xu, Jie Jiang, Sha Li, Xuechen Chen

**Affiliations:** ^1^ College of Pharmacy Jinan University Guangzhou China; ^2^ Department of Oncology Shenzhen Guangming District People's Hospital Shenzhen China

**Keywords:** clinical outcome, colorectal cancer, polygenic risk score, single nucleotide polymorphisms, survival

## Abstract

**Background:**

Several single nucleotide polymorphisms (SNPs) identified through genome‐wide association studies (GWASs) on colorectal cancer (CRC) incidence are also shown as promising predictors of clinical outcomes in CRC patients. These genetic variants might help inform precision prognostic strategies by predicting disease progression, treatment response, and overall survival, thereby guiding more personalized treatment plans. However, conflicting evidence exists regarding their clinical relevance.

**Aim:**

A systematic review and meta‐analysis was performed to assess the potential of GWAS‐identified SNPs in predicting CRC outcomes.

**Methods and Results:**

We conducted a comprehensive search of PubMed, Web of Science, Embase, and Cochrane databases up to 18 October 2024 for prospective studies that investigated the associations of CRC‐related SNPs and polygenic risk scores (PRSs) built based on multiple SNPs with clinical outcomes. Quality of the included studies was assessed using the Newcastle–Ottawa Scale, and the heterogeneity was assessed by *I* ^2^ index and Cochran's Q test. The final analysis included 22 studies with overall high quality and heterogeneity in several aspects (e.g., genetic models, ethnic background, and genetic signatures of CRC types). Among over 100 CRC risk‐related loci, 12 SNPs were statistically associated with CRC clinical outcomes (mainly survival outcomes), which were replicated in multiple studies. Notably, rs9929218 and rs6983267, located in genes involved in processes of tumorigenesis, were linked to poor survival with hazard ratios (95% CIs) of 1.26 (1.12–1.42) under a recessive model and 1.33 (1.10–1.61) under an additive model, respectively, in the stratified analysis by genetic models. Besides, PRSs built based on survival‐related SNPs were moderately associated with overall survival in CRC patients with hazard ratios exceeding 2.6 for each one‐point increase in PRS.

**Conclusions:**

Individual genetic variants and PRSs are predictive of CRC survival, and might serve as potential factors for risk stratification, which could help develop personalized treatment and surveillance strategies for CRC patients. However, the potential for false positives and the significant heterogeneity among studies that cannot be fully addressed in the current analysis due to limited data require a cautious interpretation of these findings. Large‐scale studies are warranted to further explore and validate GWAS‐identified SNPs for promising prognostic biomarkers in CRC patients while accounting for factors such as ethnic differences and tumor subtypes.

## 1 Introduction

Despite recent advances in cancer diagnostic and therapeutic modalities, colorectal cancer (CRC) remains one of the leading causes of death globally, with an estimated 1.9 million incident cases (9.6% of new cancer cases) and 0.9 million deaths (9.3% of cancer‐related deaths) in 2022 [1]. Both incidence and mortality rates are two to four times higher in countries with a high human development index compared to those with low and medium human development index. This substantial burden of CRC is partly due to the Inter‐patient and intra‐tumor heterogeneity observed among affected individuals (such as variations in the genetic factors, tumor microenvironment, drug resistance, and patient‐related factors including age, comorbidities), which contribute to the complexity of CRC prevention, treatment, and prognosis [2].

Genetic variants involved in CRC initiation have also been suggested as potential and robust predictors of clinical outcomes in CRC patients. Several studies have found that CRC risk‐related single nucleotide polymorphisms (SNPs) identified in genome‐wide association studies (GWASs) [3, 4, 5], alone or aggregated in a polygenic risk score (PRS), were also associated with the survival of CRC [6, 7, 8, 9, 10]. Understanding the prognostic impact of CRC susceptibility variants individually or as a more powerful stratification approach like PRS might aid in developing effective strategies for patient risk stratification. For example, patients with a higher genetic risk for shorter survival or increased recurrence may benefit from more aggressive treatment protocols and closer follow‐up surveillance. Additionally, identifying genetic variants associated with treatment efficacy can help tailor therapies to individual patients, ensuring that those most likely to respond to specific treatments are prioritized, thereby optimizing therapeutic outcomes. However, the evidence remains inconsistent regarding the impact of genetic variants, such as rs3217810, on CRC outcomes [9, 10]. With advances in DNA sequencing technologies and accumulated sample sizes in GWASs, more risk variants, especially those with moderate and rare effects, will be discovered, and there could be a further exploration of the role of these genetic markers in CRC prognosis and survival. Therefore, it is necessary to know what has been done and what we could improve on this topic based on the current evidence.

The objectives of this study are therefore to provide a comprehensive overview of available evidence on the associations between CRC‐related risk variants and clinical outcomes in CRC patients, and to synthesize data from eligible studies to assess whether and to what extent CRC‐related genetic variants identified through GWASs are associated with key clinical outcomes in CRC patients, as well as the potential factors that might influence these results.

## 2 Materials and Methods

This systematic review was reported according to the Preferred Reporting Items for Systematic Reviews and Meta‐Analyses (PRISMA) checklist [11]. The protocol was not registered. Ethical approval and patient‐informed consent were not required since the data presented and analyzed in this study were obtained from previously published studies.

### 2.1 Literature Search Strategies

PubMed, Embase, Web of Science, and Cochrane databases were searched from their inception to 18 October 2024. Detailed search items are provided in the Supporting Information, which were aimed at covering expressions for CRC‐related SNPs or PRSs, CRC (colon cancer, rectum cancer, or both), and clinical outcomes of patients with CRC. First, duplicates were removed, followed by the exclusion of records irrelevant to our topic after abstract review. Full texts of the remaining studies and their references were examined, and those meeting our predefined criteria were included in this review. The search strategies were independently performed by two reviewers (C.W. and J.Z.).

### 2.2 Eligibility Criteria

We included studies if they: (1) involved patients diagnosed with colon, rectum, or CRC; (2) were designed as a prospective cohort study with clinical outcomes of CRC observed during the follow‐up; (3) analyzed CRC‐related risk variants identified from previously published GWASs; in cases where the source of studied SNPs was not explicitly stated, we conducted searches on the GWAS catalog website for confirmation [12]; (4) reported effect estimates of association between CRC‐related risk variants or PRSs and clinical outcomes of CRC. Among the eligible studies meeting the criteria above, we further excluded those not conducted on humans or not published in English.

### 2.3 Data Extraction and Quality Assessment

Two reviewers (C.W. and J.Z.) independently read and extracted data from eligible studies. The following data from these studies were extracted: first author, year of publication, country, number of study participants, demographic characteristics (age and sex), types of CRC, whether a PRS was derived, number of CRC‐related SNPs, clinical outcomes of CRC, disease stage, newly diagnosed patients (yes/no), status of mismatch repair (MMR, proficient or deficient), treatment (chemotherapy, radiotherapy, or both), biosample types, genotyping methods, and genetic models. Additionally, estimated hazard ratios (HRs) and their 95% confidence intervals (CIs), along with covariates that were adjusted for, were extracted from the models in the eligible studies.

The quality of included non‐randomized studies was assessed using the Newcastle–Ottawa Scale (NOS), which consists of three domains: selection, comparability, and outcome assessment [13]. Studies were awarded stars for meeting specific criteria within each domain, with a maximum of four stars for selection, two for comparability, and three for outcome assessment. Studies with seven to nine stars were considered as studies with “low risk of bias”, otherwise, they were regarded as having a high risk of bias. Quality assessments were conducted independently by the above two authors, with disagreements resolved through discussion.

### 2.4 Data Synthesis

HRs (95% CIs) were separately pooled to assess the associations between different risk variants and clinical outcomes, and these results were visualized using forest plots. Besides, data were synthesized separately for each applied genetic model within these associations. Heterogeneity across studies was evaluated using Cochrane's Q statistic with *I* ^2^ statistic and *p*‐value [14]. If significant heterogeneity (*I* ^2^ > 50% or *p*‐value for *Q*‐statistics < 0.1) was observed, results of a random‐effects model were reported; otherwise, we kept the results of a fixed‐effects model. We presented results of both models in forest plots to show if there were any differences between them. We did not perform a meta‐analysis for the associations of PRSs with clinical outcomes in CRC patients, since the number of SNPs included in PRSs varied greatly across studies.

All analyses were conducted using R statistical software (version 4.3.1; R Foundation for Statistical Computing, Vienna, Austria). We considered all *p*‐values as two‐sided, with a significance threshold set at 0.05.

## 3 Results

### 3.1 Literature Search

**FIGURE 1:**
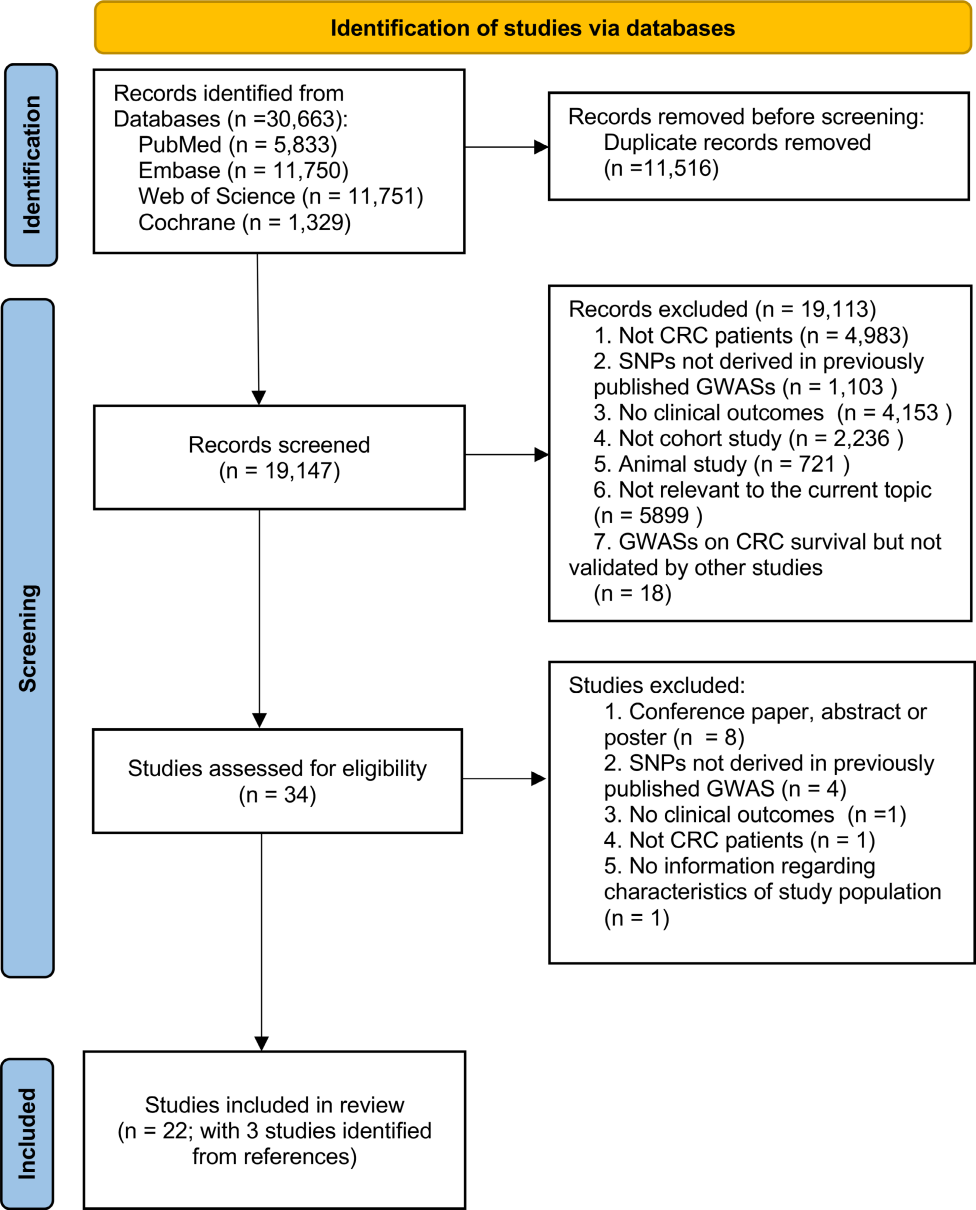
Flow diagram of inclusions of studies. Abbreviations: CRC, colorectal cancer; GWAS, genome‐wide association study; SNP, single nucleotide polymorphism.

The process of literature search is outlined in Figure 1. In brief, 19 147 records were retrieved after removing duplicates from the initial search from PubMed (*n* = 5833), Embase (*n* = 11 750), Web of Science (*n* = 11 751), and Cochrane (*n* = 1329). After exclusions, 34 records were qualified for the full‐text review. Of these, 15 studies were further excluded for not meeting the inclusion criteria. Finally, a total of 22 studies, including three [15, 16, 17] identified from cross‐referencing, were included in this review.

### 3.2 Study Characteristics

Table 1 summarizes the basic characteristics of the included studies published between 2009 and 2023 [6, 7, 8, 9, 10, 15, 16, 17, 18, 19, 20, 21, 22, 23, 24, 25, 26, 27, 28, 29, 30, 31]. Most studies were conducted in America and Europe, with five studies in Asian countries (Korea and China). Three studies had both training and validation phases [10, 27, 31]. The median/mean age of participants across studies ranged from 58 to 71 years, with data unavailable in three studies [6, 9, 17]. Regarding sex, one study exclusively enrolled female participants [19], while the remaining studies examined both sexes. Nearly all studies enrolled patients with CRC, except for two studies focusing exclusively on colon [15] or rectal cancer cases [17]. The number of SNPs analyzed varied greatly among studies, ranging from 1 to 130. PRSs were derived within five studies [6, 7, 8, 30, 31] based on SNPs identified from previously published GWASs. There were diverse clinical outcomes with a primary focus on survival‐related outcomes including overall survival/mortality, CRC‐specific survival/mortality, and disease‐free survival, followed by prognosis‐related endpoints, such as recurrence, radiographic response rate, and time to progression. Most studies (*n* = 19) found statistically significant associations (*p* < 0.05) between CRC‐related SNPs/PRS and clinical outcomes in CRC patients. DNA was mainly extracted from blood samples and genotyped using Taqman and array‐based sequencing methods (Table S1). Results for the quality of included studies are summarized in Table S2. Overall, the risk of bias in most studies (*n* = 21) was rated low (the median NOS score was 8 with the interquartile range of 7–9, and over half of the studies were scored 9 stars), while one study [15] was considered to have a high risk of bias mainly due to the potential selection bias.

**TABLE 1:** Characteristics of studies included in the review.

Study ref.		Country	Patients			CRC types	SNP, *N*	PRS	Outcomes	SNP/PRSs associated with outcomes of CRC
			Total, *N*	Age(y), mean (SD)/median (age range)	Male, *N* (%)					
Cicek [15]		US/Canada	460	62 (10)	249 (54)	CC	4	N	5‐year DFS/OS	N
Tenesa [18]		UK	2838	61 (11)	1630 (57)	CRC	10	N	All‐cause mortality/CRC‐specific mortality	N
Passarelli [19]		US	727	50–74	0	CRC	2	N	CSS/OS	N
Xing [20]		China	351	60 (/)	194 (55)	CRC	7	N	Recurrence/CRC‐specific mortality	Y
Dai [21]		US	285	58 (/)	179 (63)	CRC	26	N	OS/recurrence	Y
Hoskins [22]		US	583	65 (26–93)	304 (52)	CRC	11	N	OS	Y
Phipps [6]		US	2611	/	698 (27)	CRC	16	Y	OS/CSS	Y
Abulí [16]		Spain	1235	71 (/)	733 (59)	CRC	16	N	DFS/OS/RFI	Y
Sanoff [23]		US/Puerto Rico/Canada/South Africa	524	61 (28–88)	309 (59)	CRC	6	N	RR/TTP/OS	Y
Kang [24]		Korea	776	64 (21–89)	432 (56)	CRC	22	N	DFS/OS	Y
Morris [25]		UK	4327	59 (8)	2570 (59)	CRC	19	N	5‐year OS	Y
Savas [26]		Canada	505	61 (21–75)	307 (61)	CRC	102	N	DFS/OS	Y
Smith [27]	Training	UK	2078	62 (10)	1371 (66)	CRC	20	N	OS	Y
	Validation	UK/US	5552	67 (/)	2237 (40)		3			Y
Noci [28]		Italy	733	64 (24–93)	428 (58)	CRC	86	N	OS/TTR	Y
Wang [7]		China	731	67 (9)	241 (33)	CRC	38	Y	All‐cause mortality/CRC‐specific mortality	Y
Hu [17]		Germany	230	/	/	RC	24	N	Recurrence/OS	Y
Song [8]		Korea	1374	58 (12)	856 (62)	CRC	30	Y	DFS/OS	Y
Wang [29]		China	125	Median: 59	71 (57)	CRC	1	N	PFS/RR	Y
He [30]		UK	5675	65 (IQR: 55–72)	3235 (57)	CRC	130	Y	OS/CSS	Y
Summers [9]		UK	1948	/	/	CRC	76	N	OS	Y
He [10]	Discovery	UK	5675	65 (IQR: 55–72)	3235 (57)	CRC	129	N	OS/CSS	Y
	Validation	UK	2474	65 (IQR: 61–70)	1035 (42)	CRC	2			Y
Xin [31]	UK Biobank	UK	2621	65 (7)	1555 (59)	CRC	88	Y	OS/CSS	Y
	TCGA	European countries	470	67 (13)	248 (53)	CRC	81			N

*Note:* “/” for N/A.

Abbreviations: CC, colon cancer, CRC, colorectal cancer; CSS, CRC‐specific survival; DFS, disease‐free survival; IQR, interquartile range; N, no; OS, overall survival; PFS, progression‐free survival; PRS, polygenic risk score; RC, rectal cancer; RFI, recurrence‐free interval; RR, radiographic response rate; SNP, single nucleotide polymorphism; TCGA, The Cancer Genome Atlas Program; TTP, time to progression; TTR, time to recurrence; Y, yes.

### 3.3 Clinical Characteristics of CRC Patients in Included Studies

One study [31] did not present the clinical characteristics of CRC patients, and thus only 21 studies were included in Table S3. Among the eligible studies, patients diagnosed with CRC in stage II/III constituted the main study population, with two studies (the training phase of the study by Smith et al. [27] and the study by Summers et al. [9]) focusing exclusively on stage IV patients. Most studies (*n* = 20) recruited newly diagnosed patients to minimize survival bias. Only four studies [15, 16, 19, 26] provided data on MMR status. Chemotherapy was the primary treatment for CRC patients in the included studies with available information on treatment (*n* = 15).

### 3.4 CRC‐Related SNPs and Clinical Outcomes in CRC Patients

Analyzed SNPs and those significantly associated (*p* < 0.05) with CRC outcomes in the included studies are listed in Tables S4 and S5. Among these associations, 12 SNPs (rs9929218, rs10795668, rs16892766, rs6983267, rs7495132, rs10161980, rs4939827, rs10749971, rs174537, rs3087967, rs3217810, rs35509282) were identified to be associated with clinical outcomes of CRC in at least two studies, as presented in Table S6. Additionally, for meta‐analysis purposes, statistically insignificant associations between these SNPs and CRC outcomes are also presented there.

**FIGURE 2:**
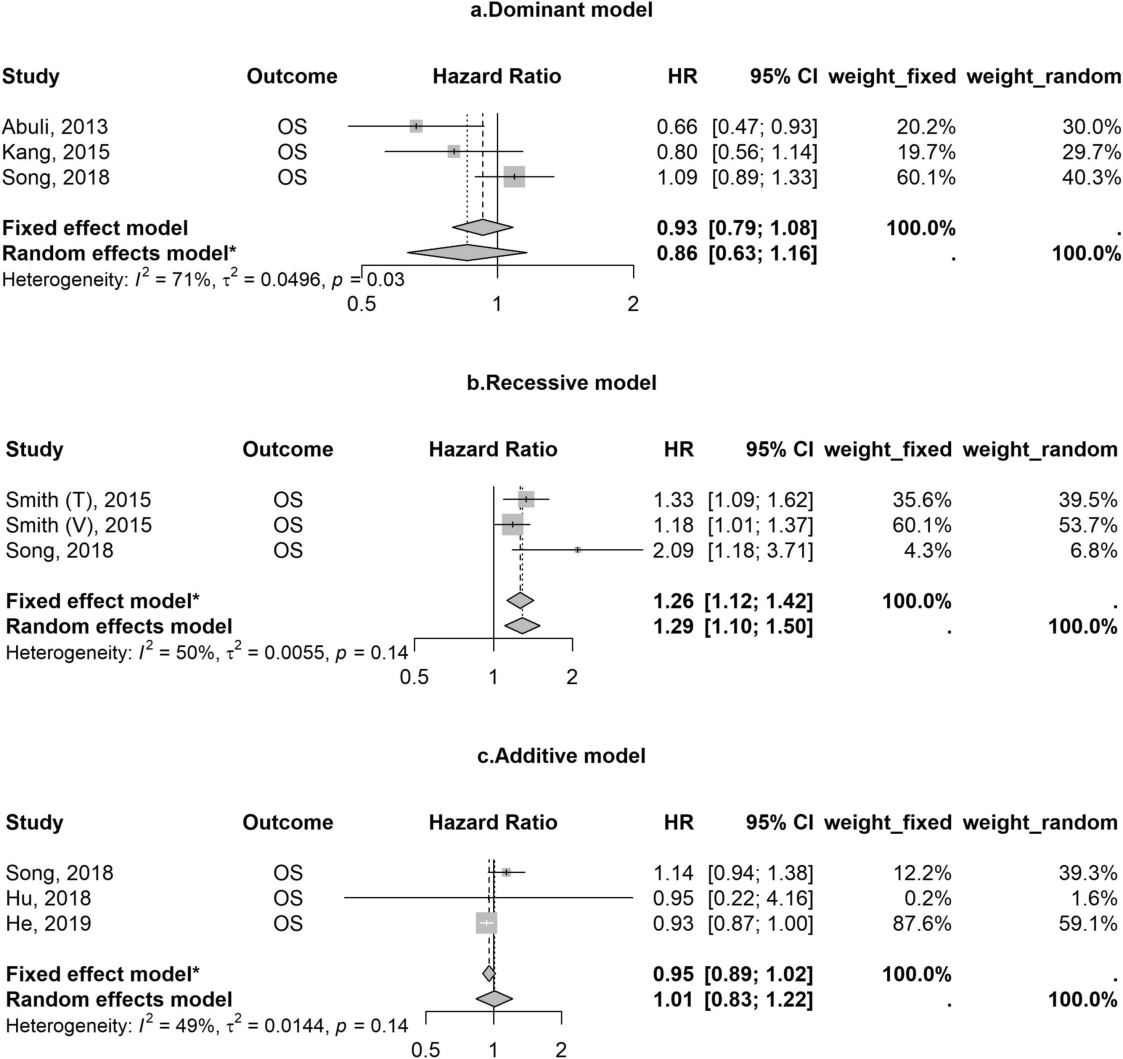
Associations of rs9929218 with overall survival in patients with colorectal cancer. Abbreviations: CI, confidence interval; HR, hazard ratio; OS, overall survival; T, training; V, validation. *The model used is according to the heterogeneity across studies.

**FIGURE 3:**
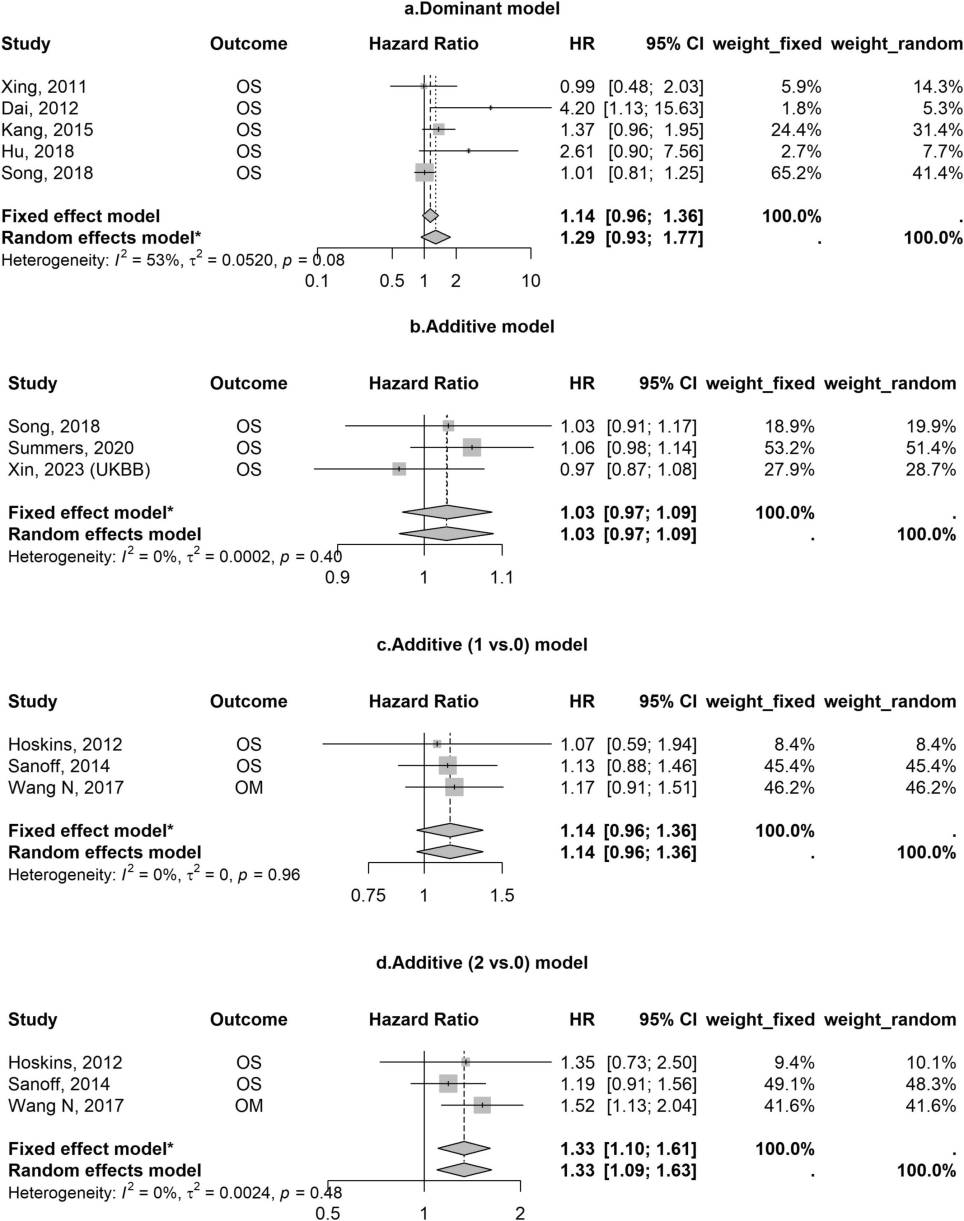
Associations of rs6983267 with overall survival/mortality in patients with colorectal cancer. Abbreviations: CI, confidence interval; HR, hazard ratio; OM, overall mortality; OS, overall survival. *The model was used according to the heterogeneity across studies.

We synthesized the results for SNPs (rs9929218, rs10795668, rs16892766, rs6983267, rs4939827, and rs3087967) with the same outcome based on the same genetic model in multiple studies (*n* ≥ 3). Patients with the minor allele of rs9929218 had worse overall survival compared to those without it under recessive models (fixed effect model: HR 1.26; 95% CI 1.12–1.42), but not under dominant or additive models (Figure 2). rs6983267 was also associated with poor survival under additive models (fixed effect model: 2 vs. 0 minor allele: HR 1.33; 95% CI 1.10–1.61, Figure 3). Meta‐analyses of other SNPs did not yield significant results (Figures S1–S4). Results were robust and varied a little by using different synthesis models. The SNP rs10161980 was associated with CRC‐specific survival in the discovery and validation datasets in the study by He et al. [10], which remained statistically significant after multiple corrections. However, a meta‐analysis for the associations between rs10161980 and CRC‐specific survival in CRC patients was not conducted due to the limited evidence.

### 3.5 CRC‐Related PRS and Clinical Outcomes in CRC Patients

We observed that PRSs based on SNPs related to CRC initiation were not reliable predictors of clinical outcomes of CRC [6, 8, 30, 31]. However, PRSs that summed alleles related to both CRC incidence and survival showed moderate associations with CRC survival (HRs for the highest level of PRS vs. the lowest ranged from 1.34 to 1.60), which were independent of important confounders such as age and stage [6, 7, 8] (see Table 2).

**TABLE 2:** Associations of polygenic risk score with clinical outcomes of colorectal cancer.

Study ref.		SNP, *N*	SNP associated with survival	Weighted PRS	PRS levels	aHR (95% CI)	Adjustment	Outcome
Phipps [6]		16	N	N	Per allele increase	0.96 (0.91–1.01)	Age, sex, and stage	OS
					Per allele increase	0.98 (0.93–1.03)		CSS
		16	Y	N	Per allele increase	1.06 (1.02–1.10)		OS
					Per allele increase	1.08 (1.04–1.13)		CSS
		3	Y	N	Per allele increase	1.13 (1.06–1.20)		OS
					Per allele increase	1.14 (1.05–1.23)		CSS
Wang [7]		5	Y	Y	Quintile 5 vs. Quintile 1	1.50 (1.06, 2.12)	Age, sex, tumor, location, BMI, and stage	CRC‐specific mortality
					Quintile 5 vs. Quintile 1	1.51 (1.09–2.10)		All‐cause mortality
Song [8]		5	Y	N	Q4 vs. Q1	1.34 (1.06–1.69)	Age at diagnosis and TNM stage	DFS
					Per allele increase	1.08 (1.02–1.15)		
					Q4 vs. Q1	1.51 (1.17–1.95)		OS
					Per allele increase	1.11 (1.04–1.18)		
			Y	Y	Q4 vs. Q1	1.41 (1.12–1.78)		DFS
					Per one point increase	2.66 (1.48–4.79)		
					Q4 vs. Q1	1.39 (1.09–1.77)		OS
					Per one point increase	2.76 (1.55–4.89)		
		2	Y	N	Q3 vs. Q1	1.38 (1.06–1.81)	Age at diagnosis and TNM stage	DFS
					Q4 vs. Q1	1.53 (1.14–2.05)		
					Per allele increase	1.13 (1.04–1.23)		
					Q3 vs. Q1	1.39 (1.03–1.87)		OS
					Q4 vs. Q1	1.58 (1.14–2.18)		
					Per allele increase	1.15 (1.05–1.26)		
			Y	Y	Q3 vs. Q1	1.34 (1.04–1.74)		DFS
					Q4 vs. Q1	1.49 (1.12–1.99)		
					Per one point increase	2.63 (1.37–5.06)		
					Q3 vs. Q1	1.40 (1.02–1.93)		OS
					Q4 vs. Q1	1.60 (1.13–2.25)		
					Per one point increase	2.67 (1.38–5.14)		
		17	N	N	Per allele increase	0.98 (0.96–1.01)	Age at diagnosis, and TNM stage	DFS
					Per allele increase	0.98 (0.96–1.01)		OS
			N	Y	Per one point increase	0.92 (0.79, 1.06)		DFS
					Per one point increase	0.90 (0.77, 1.05)		OS
He [30]		130	N	N	Per allele increase	1.00 (0.95–1.04)	Age at diagnosis, sex, and AJCC stage	OS
						1.03 (0.97–1.08)		CSS
Xin, 2023 [31]	UK Biobank	88	N	Y	Per SD increase	0.93 (0.87–1.00)^a^	Sex, age, BMI, smoking status, drinking status, and first 10 principal components	OS
						0.96 (0.89–1.05)^a^		CSS
						1.53 (1.41–1.67)		CRC‐specific mortality
						0.94 (0.87–1.02)^a^		3‐year OS
						0.97 (0.89–1.07)^a^		3‐year CSS
						0.93 (0.87–1.00)^a^		5‐year OS
						0.97 (0.89–1.05)^a^		5‐year CSS
	TCGA	81	N	Y	Per SD increase	1.10 (0.90–1.36)^a^	Sex, age, and first 10 principal components	OS
						1.41 (1.06–1.87)^a^		CSS
						1.02 (0.81–1.29)		3‐year OS
						1.18 (0.87–1.59)^a^		3‐year CSS
						1.05 (0.85–1.30)^a^		5‐year OS
						1.32 (0.99–1.76)^a^		5‐year CSS

*Note:* Weighted PRS was calculated as a sum of the number of risk alleles multiplied by corresponding genotype effect size estimates derived from genome‐wide association studies. Unweighted PRS was calculated as a sum of the number of risk alleles.

Abbreviations: aHR, adjusted hazard ratio; BMI, Body Mass Index; CRC, colorectal cancer; CSS, CRC‐specific survival; DFS, disease‐free survival; N, No; OS, overall survival; PRS, polygenic risk score; Q, quartile; SD, standard deviation; TCGA, The Cancer Genome Atlas; TNM, tumor node metastasis; Y, yes.

^a^ Not statistically significant after multiple corrections.

## 4 Discussion

This review provides an overview of a total of 22 studies investigating the associations of individual or aggregated CRC‐related SNPs identified by GWASs with clinical outcomes among patients with CRC. Over the past two decades, there has been an extensive exploration of this topic with the updated results from GWASs on CRC risk. Among the examined associations, the associations of rs9929218 and rs6983267 with overall survival in CRC patients demonstrated robustness and low heterogeneity in the meta‐analysis. Besides, PRSs aggregating survival‐related SNPs were moderately associated with CRC survival. Despite some promising results, findings from included studies might suffer from the issue of false positives due to the lack of multiple corrections, and substantial heterogeneity across studies requires careful consideration in the interpretation of results.

Our meta‐analysis showed that rs9929218 was associated with poor survival among studied CRC susceptibility genetic variants using recessive genetic models rather than others. The additive model, which assumes a linear relationship between each allele and cancer outcomes, is commonly employed in genetic association studies. However, this approach might miss the variants with a true mode of inheritance being recessive or other in some studies [17, 30]. Our results underscore the importance of carefully selecting genetic models in analyses. rs9929218 is located in the intron of gene CDH1 and in strong linkage disequilibrium with rs16260 in the CDH1 promoter, which downregulates the expression of the gene CDH1 that encodes E‐cadherin [27, 32]. Loss of function of this gene is linked to tumor progression and metastasis [33]. Additionally, rs6983267, which influences CRC risk through the Wnt signaling pathway [34], was also associated with poor survival in this meta‐analysis. Subgroup analyses by genetic models in the current analyses also revealed that this association was statistically strong under the additive model rather than the dominant model. Although Dai et al. reported a strong association between rs6983267 and overall survival under the dominant model (HR 4.20, 95% CI 1.13–15.63), their results were likely limited by insufficient statistical power and low precision [21]. Evidence supporting the associations of other SNPs with clinical outcomes in CRC patients is currently limited and not well replicated (see Table S6). One of the possible explanations is that SNPs discovered in GWASs may not fully capture the biological mechanisms underlying CRC development, since most of them are tag SNPs located in non‐protein regions in linkage disequilibrium with causal SNPs [35]. Identifying causal SNPs linked to the SNPs discovered in GWAS loci might help find strong predictors of CRC outcomes and new therapeutic targets [35, 36].

Interestingly, the associations of rs9929218 with overall survival seem to be stronger in CRC patients from Korea [8] than those from the UK/US [27], which is opposite to the results regarding its associations with CRC risk [37]. These findings underscore the importance of considering distinctions in ethnic backgrounds in such studies. Although race was added as an adjustment in several studies [19, 21, 23], race‐specific results were not reported due to limited sample sizes. Besides, the limited number of studies on the same SNPs in this review limited our ability to perform further analyses by race. The issue of ethnic differences might also exist in the meta‐analyses for other examined SNPs. For example, for the association between rs10795668 and overall survival, results were inconsistent between the studies from Korea [8, 24] and the study from Spain [16] (see Figure S1), which might have led to the non‐significant synthesized estimate of risk (HR 0.98, 95% CI 0.70–1.39) under the random effects model.

Although stage, a key clinical determinant for patients' survival [38], was well controlled in the models, other prognosis‐related data such as MMR status were lacking in most included studies, which might have partly contributed to the heterogeneous results within the analyzed SNPs. MMR serves as a robust prognostic and predictive molecular marker, even within the same CRC stage [39]. MMR deficiency can lead to microsatellite instability (MSI), a molecular phenotype that was associated with a better prognosis in CRC patients compared to those with microsatellite stable (MSS) tumors [40]. MMR status could have an impact on the outcomes of the included studies, but its influence is relatively modest due to its relatively low prevalence in CRC patients (approximately 15%–20% of sporadic CRC [41]).

Due to substantial variations in the number and overlap of SNPs included in the PRSs across studies, we refrained from conducting a meta‐analysis of the associations between PRS and clinical outcomes in CRC patients. Combining such heterogeneous data could potentially yield misleading conclusions. However, as more studies with standardized PRS construction methods and consistent clinical endpoints are published, future meta‐analyses may become more feasible and reliable. PRSs aggregating survival‐related SNPs appear to be a promising predictor of survival in CRC patients. In the study by Song et al. [8], PRSs constructed based on two and five SNPs were moderately associated with disease‐free and overall survival in CRC patients. Although these findings have not been validated in other populations, they highlighted the potential use of PRSs in predicting CRC outcomes.

It should be noted that survival‐related PRSs presented in the current study primarily rely on a low number of SNPs. Therefore, their predictive performance might be suboptimal due to the limited SNP coverage and incomplete heritability capture. With more survival‐related risk variants identified in GWASs that examine the role of inherited variation in patient outcomes, PRSs might become a powerful predictive approach for CRC outcomes. However, such GWASs are often challenged with limited statistical power due to small sample sizes, making it difficult to detect rare variants or variants with modest effects [42, 43]. In this case, a well‐designed study based on a large sample size and a combination of SNPs along with detailed treatment options might help identify robust predictive genetic markers to inform treatment decisions for CRC patients [44, 45, 46]. For example, a recently published study reported that rs1234556 on chromosome 1, rs11052270 on chromosome 12, and rs11858406 on chromosome 15 were associated with differential survival between patients who received oxaliplatin‐based chemotherapy and patients receiving other regimens [46]. Moreover, PRS developed specifically for CRC risk are limited in their ability to account for the variability in disease status and the heterogeneity of disease subtypes, and the influence of lifestyle factors, which are well associated with patient survival outcomes [41]. By integrating these critical factors with PRS as a composite metric, a more comprehensive understanding of CRC prognosis might be achieved, potentially leading to more tailored and effective clinical interventions.

Our study has several strengths, including comprehensive search strategies and well‐defined eligibility criteria to identify relevant papers for the topic of interest. Besides, we extracted detailed data to characterize the evidence to date to inform further research in this field. However, several limitations should also be addressed. First, despite our efforts to include all pertinent evidence, the possibility of missing relevant studies remains, such as those published in non‐English languages, which could have introduced language bias. However, the impact might be minimal, since only quite a small proportion (< 3%) of non‐English papers were excluded during paper selection, and the actual number is likely lower when accounting for duplicates across databases. Limiting systematic reviews to English‐only is common. However, with accumulated data on GWASs in other populations and improvement in translation accuracy, future research should incorporate data without language limitation to enhance the generalizability of findings and reduce potential language and population biases. Second, one study was excluded because the author did not provide any detailed information except the HRs of associations of interest [47], possibly leading to outcome‐reporting bias [48]. Third, the synthesized results might be biased since we were unable to perform subgroup analyses by possible causes of heterogeneity due to the limited data available.

In conclusion, studies to date with overall high quality have shown some promising results that were supported by reasonable biological mechanisms underlying cancer development. However, most other findings from included studies with high heterogeneity were not well replicated and could have suffered from an issue of false positives. Large and well‐designed prospective studies are warranted to explore and validate the promising SNPs, particularly the survival‐related SNPs or causal SNPs involved in tumorigenesis, or their combination with treatment or other biomarkers to predict clinical outcomes among CRC patients in the whole and in subgroups by important confounders of CRC outcomes.

## Author Contributions

**Chengmi Wu:** data curation; formal analysis; methodology; writing – review and editing. **Jingyi Zhou:** data curation; formal analysis; methodology; writing – review and editing. **Qian Wu:** writing – review and editing. **Shu Xu:** supervision; writing – review and editing. **Jie Jiang:** supervision; writing – review and editing. **Sha Li:** conceptualization; project administration; supervision; writing – review and editing. **Xuechen Chen:** conceptualization; project administration; supervision; funding acquisition; writing – original draft; writing – review and editing. All authors provided comments, revised the draft, and approved the final version of the manuscript.

## Ethics Statement

The authors have nothing to report.

## Conflicts of Interest

The authors declare no conflicts of interest.

## Supporting information


**Data S1.** Supporting Information.

## Data Availability

Data analyzed in this review was publicly available. Analytic code is avaiable from corresponding author upon reasonable request.
