# Supplementary material for: Colorectal Cancer Risk Loci: Prognostic Factors for Clinical Outcomes? A Systematic Review and Meta‐Analysis
Source: Cancer Rep (Hoboken). 2025 May 19;8(5):e70230. doi: 10.1002/cnr2.70230 (PMC12931425; doi:10.1002/cnr2.70230)
Supplement: Supplementary file 1 — Data S1. Supporting Information. [file CNR2-8-e70230-s001.zip › cnr270230-sup-0002-Supinfo2@Supplementary methods.docx]

**Searching strategies**

**PubMed:**

#1:

(genetic* [title/abstract] OR locus [title/abstract] OR loci [title/abstract] OR variant* [title/abstract] OR polymorphism* [title/abstract] OR SNP [title/abstract] OR SNPs [title/abstract] OR “polygenic risk score*” [title/abstract] OR PRS [title/abstract] OR PRSs [title/abstract] OR “polygenic score*” [title/abstract] OR “genetic risk score*” [title/abstract] OR GRS [title/abstract] OR GRSs [title/abstract])

#2:

((CRC [title/abstract] OR colorect* [title/abstract] OR rectal [title/abstract] OR rectum [title/abstract] OR colon [title/abstract] OR “large bowel” [title/abstract]) AND (carcinoma* [title/abstract] OR cancer [title/abstract] OR neoplas* [title/abstract] OR malignan* [title/abstract] OR adenocarcinoma* [title/abstract])) OR pan-cancer [title/abstract]

#3:

(surviv* [title/abstract] OR mortality [title/abstract] OR death [title/abstract] OR prognosis [title/abstract] OR “long term” [title/abstract] OR “period analysis” [title/abstract])

#1 AND #2 AND #3

Filters: Humans, English

**Web of Science:**

#1:

TS=((genetic* OR locus OR loci OR variant* OR polymorphism* OR SNP OR SNPs OR “polygenic risk score*” OR PRS* OR PRSs OR “polygenic score*” OR “genetic risk score*” OR GRS OR GRSs))

#2:

(TS=((CRC OR colorect* OR rectal OR rectum OR colon OR “large bowel” ) AND (carcinoma* OR cancer OR neoplas* OR malignan* OR adenocarcinoma*)) OR TS=(pan-cancer))

#3:

TS=((surviv* OR mortality OR death OR prognosis OR “long term” OR “period analysis”)

#4：

TI= ((animal* OR “animal experiment*” OR “animal model” OR “animal tissue” OR “non human” OR nonhuman OR rat OR rats OR mice OR mouse OR swine OR porcine OR murine OR sheep OR lambs OR pig OR pigs OR piglets OR rabbit OR rabbits OR cat OR cats OR dog OR dogs OR cattle OR bovine OR monkey OR monkeys OR trout OR marmoset*) )

((#1 AND #2AND #3 NOT#4) AND LANGUAGE: (english)

**Embase**

#1: genetic*:ti,ab,kw OR locus:ti,ab,kw OR loci:ti,ab,kw OR variant*:ti,ab,kw OR polymorphism*:ti,ab,kw OR SNP:ti,ab,kw OR SNPs:ti,ab,kw OR 'polygenic risk score*':ti,ab,kw OR PRS*:ti,ab,kw OR PRSs:ti,ab,kw OR 'polygenic score*':ti,ab,kw OR 'genetic risk score*':ti,ab,kw OR GRS:ti,ab,kw OR GRSs:ti,ab,kw

#2: ((CRC:ti,ab,kw OR colorect*:ti,ab,kw OR rectal:ti,ab,kw OR rectum:ti,ab,kw OR colon:ti,ab,kw OR 'large bowel':ti,ab,kw) AND (carcinoma*:ti,ab,kw OR cancer:ti,ab,kw OR neoplas*:ti,ab,kw OR malignan*:ti,ab,kw OR adenocarcinoma*:ti,ab,kw)) OR (pan-cancer:ti,ab,kw)

#3:surviv*:ti,ab,kw OR mortality:ti,ab,kw OR death:ti,ab,kw OR prognosis:ti,ab,kw OR 'long term':ti,ab,kw OR 'period analysis':ti,ab,kw

(#1 AND #2) AND #3 AND [english]/lim

Filtered by: human study

**Cochrane**

#1: (genetic*):ti,ab,kw OR (locus):ti,ab,kw OR (loci):ti,ab,kw OR (variant*):ti,ab,kw OR (polymorphism*):ti,ab,kw OR (SNP):ti,ab,kw OR (SNPs):ti,ab,kw OR ('polygenic risk score*'):ti,ab,kw OR (PRS*):ti,ab,kw OR (PRSs):ti,ab,kw OR ('polygenic score*'):ti,ab,kw OR ('genetic risk score*'):ti,ab,kw OR (GRS):ti,ab,kw OR (GRSs):ti,ab,kw

#2: ((((CRC):ti,ab,kw OR (colorect*):ti,ab,kw OR (rectal):ti,ab,kw OR (rectum):ti,ab,kw OR (colon):ti,ab,kw OR ('large bowel'):ti,ab,kw) AND ((carcinoma*):ti,ab,kw OR (cancer):ti,ab,kw OR (neoplas*):ti,ab,kw OR (malignan*):ti,ab,kw OR (adenocarcinoma*):ti,ab,kw))) OR ((pan-cancer):ti,ab,kw)

#3:(surviv*):ti,ab,kw OR (mortality):ti,ab,kw OR (death):ti,ab,kw OR (prognosis):ti,ab,kw OR ('long term'):ti,ab,kw OR ('period analysis'):ti,ab,kw

#1 AND #2 AND #3

Filters: English
