# Supplementary material for: Colorectal Cancer Risk Loci: Prognostic Factors for Clinical Outcomes? A Systematic Review and Meta‐Analysis
Source: Cancer Rep (Hoboken). 2025 May 19;8(5):e70230. doi: 10.1002/cnr2.70230 (PMC12931425; doi:10.1002/cnr2.70230)
Supplement: Supplementary file 1 — Data S1. Supporting Information. [file CNR2-8-e70230-s001.zip › cnr270230-sup-0004-FigureS1-S4@Supplementary Figures1216.docx]

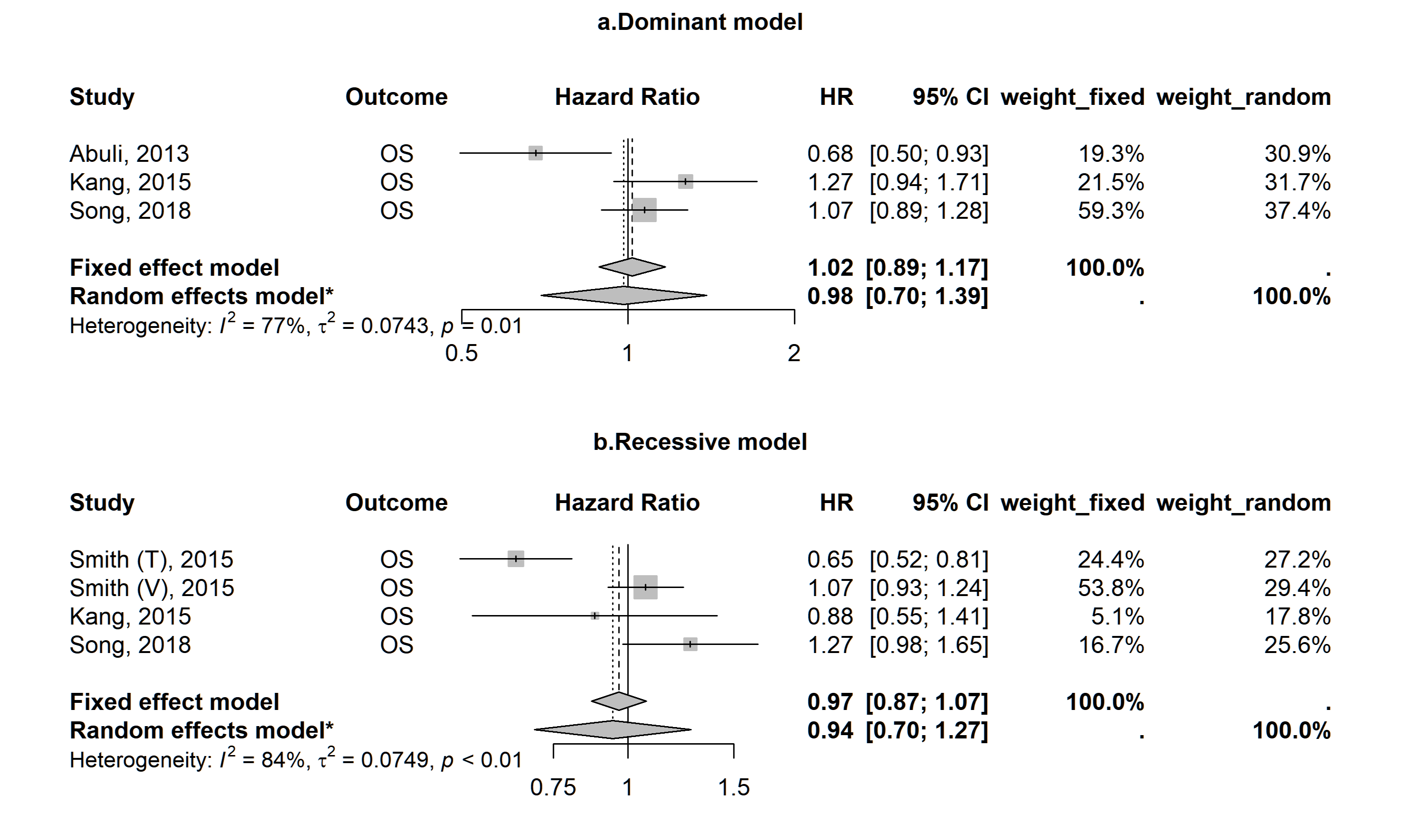


**Figure S1.** Associations of rs10795668 with overall survival in patients with colorectal cancer.

**Abbreviations:** CI, confidence interval; HR, hazard ratio; OS, overall survival**;** T, training; V, validation.

*The model used according to the heterogeneity across studies.


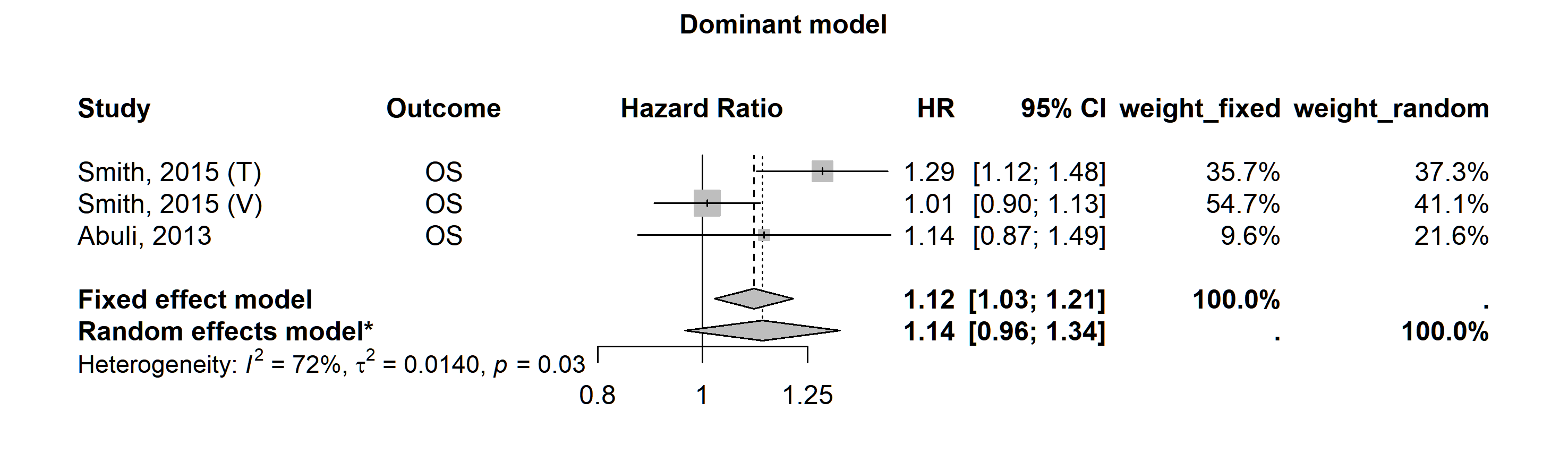


**Figure S2.** Associations of rs16892766 with overall survival in patients with colorectal cancer.

**Abbreviations:** CI, confidence interval; HR, hazard ratio; OS, overall survival**;** T, training; V, validation.

*The model used according to the heterogeneity across studies.


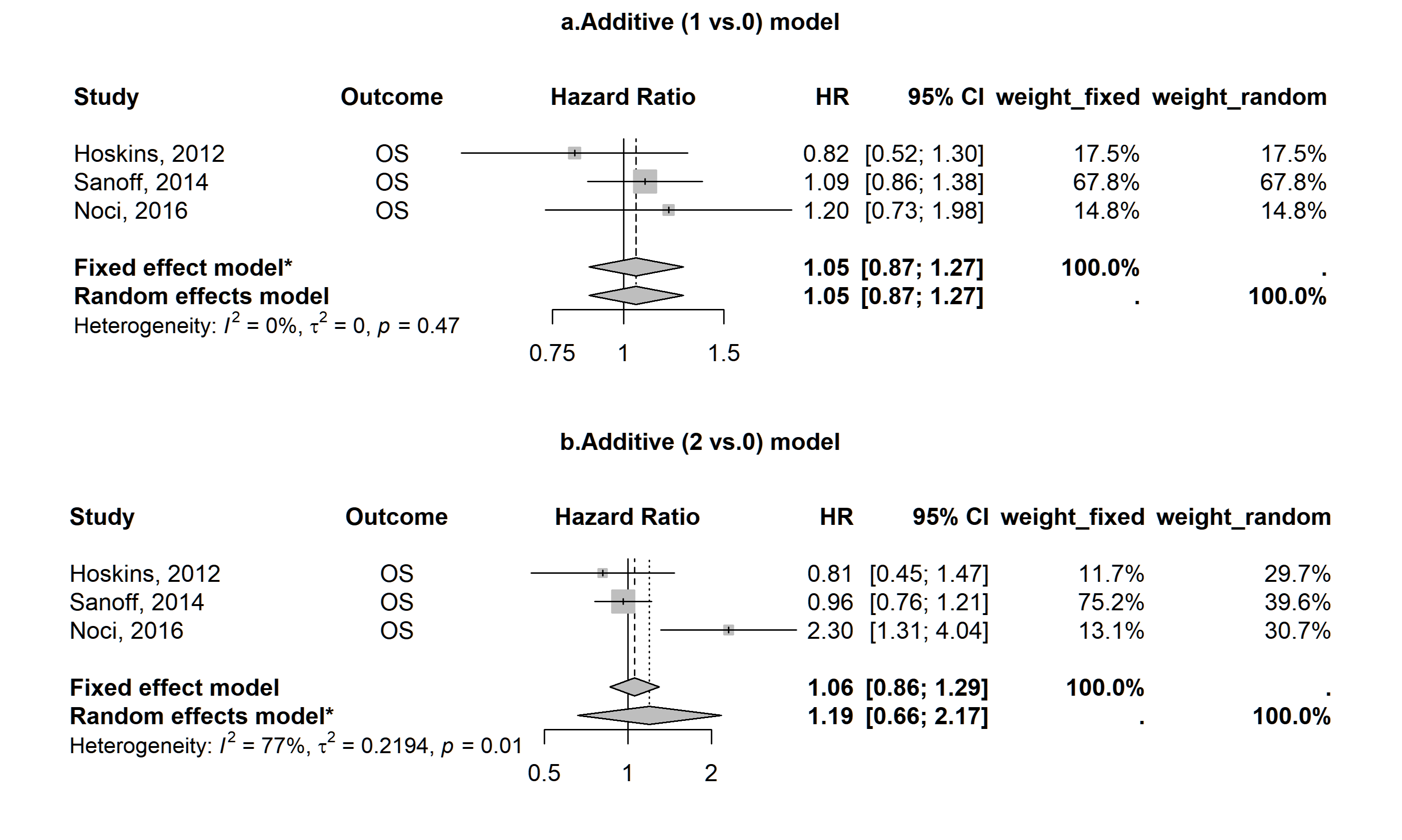


**Figure S3.** Associations of rs4939827 with overall survival in patients with colorectal cancer.

**Abbreviations:** CI, confidence interval; HR, hazard ratio; OS, overall survival**.**

*The model used according to the heterogeneity across studies.

**
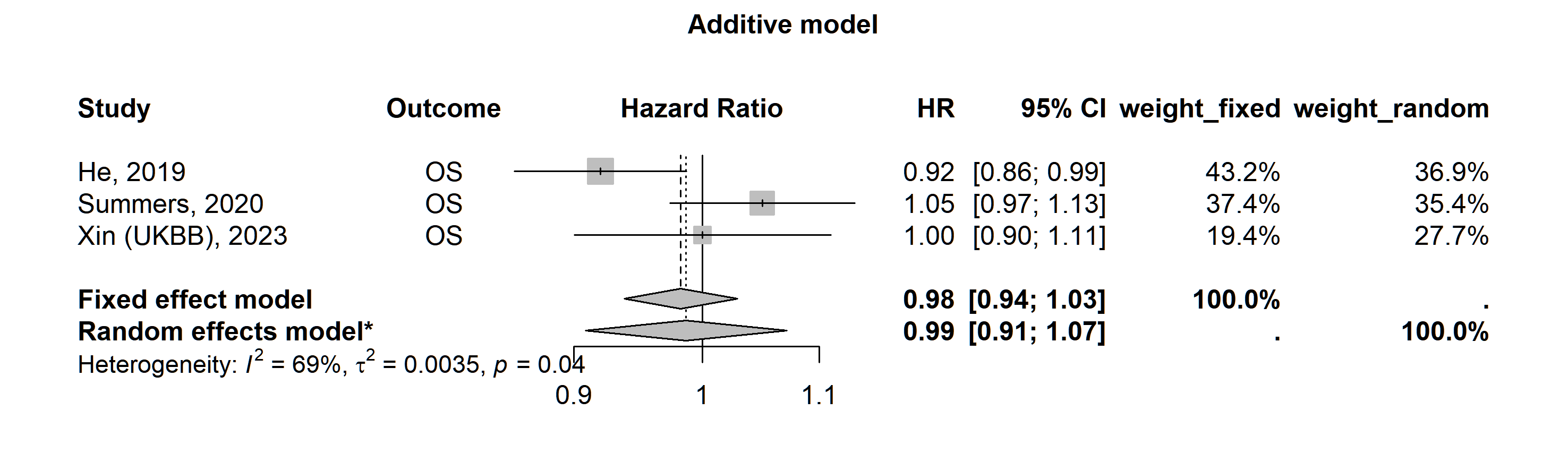
**

**Figure S4.** Associations of rs3087967 with overall survival in patients with colorectal cancer.

**Abbreviations:** CI, confidence interval; HR, hazard ratio; OS, overall survival; UKBB, UK Biobank.

*The model used according to the heterogeneity across studies.
